# Ecological momentary interventions for mental health: A scoping review

**DOI:** 10.1371/journal.pone.0248152

**Published:** 2021-03-11

**Authors:** Andreas Balaskas, Stephen M. Schueller, Anna L. Cox, Gavin Doherty

**Affiliations:** 1 School of Computer Science and Statistics, Trinity College Dublin, Dublin, Ireland; 2 Department of Psychological Science, University of California, Irvine, Irvine, CA, United States of America; 3 UCLIC, University College London, London, United Kingdom; South African Medical Research Council, SOUTH AFRICA

## Abstract

**Background:**

The development of mobile computing technology has enabled the delivery of psychological interventions while people go about their everyday lives. The original visions of the potential of these “ecological momentary interventions” were presented over a decade ago, and the widespread adoption of smartphones in the intervening years has led to a variety of research studies exploring the feasibility of these aspirations. However, there is a dearth of research describing the different dimensions, characteristics, and features of these interventions, as constructed.

**Objective:**

To provide an overview of the definitions given for “ecological momentary interventions” in the treatment of common mental health disorders, and describe the set of technological and interaction possibilities which have been used in the design of these interventions.

**Methods:**

A systematic search identified relevant literature published between 2009 and 2020 in the PubMed, PsycInfo, and ACM Guide to the Computing Literature databases. Following screening, data were extracted from eligible articles using a standardized extraction worksheet. Selected articles were then thematically categorized.

**Results:**

The search identified 583 articles of which 64 met the inclusion criteria. The interventions target a range of mental health problems, with diverse aims, intervention designs and evaluation approaches. The studies employed a variety of features for intervention delivery, but recent research is overwhelmingly comprised of studies based on smartphone apps (30 of 42 papers that described an intervention). Twenty two studies employed sensors for the collection of data in order to provide just-in-time support or predict psychological states.

**Conclusions:**

With the shift towards smartphone apps, the vision for EMIs has begun to be realised. Recent years have seen increased exploration of the use of sensors and machine learning, but the role of humans in the delivery of EMI is also varied. The variety of capabilities exhibited by EMIs motivates development of a more precise vocabulary for capturing both automatic and human tailoring of these interventions.

## 1 Introduction

Psychological disorders are characterized by impairments in thoughts, feelings and behaviors, and are a leading global cause of disability [1]. Mental health problems will affect one third of the population worldwide during their lifetime [2], however only 20% of those affected seek treatment [3]. The economic barriers to the delivery of treatment in traditional clinical settings are daunting [4]. Because of this, there is much interest in the development of new forms of treatment for common mental health conditions.

Technology advances have created new opportunities for the delivery of treatment. Support for real-time data-capture through mobile phones has created opportunities for the observation of behaviors in patients daily lives. This in turn opens up new opportunities for the delivery of interventions in everyday life. This embedding of interventions into people’s daily lives has been called “ecological momentary intervention” (EMI). Such interventions are ecological in that they are delivered in the context of one’s life and they are momentary as they tend to be brief and responsive to the “moment” [5].

### 1.1 Defining ecological momentary interventions

The term “ecological momentary intervention” was first coined in 2005 by Patrick et al. [6]. The authors presented an ecological framework for cancer communication and discussed the possible contribution of ecological momentary assessments (EMA) to cancer communication. They envisioned delivering tailored interventions based on EMA responses [6]. The capabilities of such an “ecological momentary intervention” should include the provision of personalized feedback based on real-time assessment responses and even environmental data or other contextual factors [6].

Heron and Smyth [5] provided a concise definition for the term ecological momentary interventions as “treatments that are provided to people during their everyday lives (i.e. in real time) and in natural settings (i.e. real world)” [5]. Treatment is delivered using mobile technology, and can be implemented standalone or as a supplement to existing treatment [5].

The use of these systems has been described as a “therapist in your pocket” approach, and is widely viewed to possess “the potential to revolutionize clinical treatment” [7]. The argument is that these systems provide the capability to deliver psychological intervention strategies at opportune moments, in real-world settings, and in an accessible and scalable fashion.

### 1.2 Initial explorations

An influential review by Heron and Smyth in 2010 synthesized and critiqued mobile technology-based EMIs delivered through the use of palmtop computers and mobile phones [5]. Twenty-seven interventions were reviewed targeting a variety of psychological, behavioral, and physical issues. The review described the qualities of EMI including delivery methods, intervention components, the content of EMI and its tailoring. At that point in time, interventions were delivered using a variety of technologies including SMS, palmtop computers and mobile phone voice calling capabilities. The majority of the EMI systems they reviewed were provided in conjunction with interactive websites, or were used to supplement group or individual psychotherapy.

EMIs tailored intervention messages either with information obtained from pre-intervention assessments, or by delivering interventions at predefined times when individuals were in need of additional support. EMI prompting protocols differed between studies, and the prompts were delivered either at fixed times, at random times, or at tailored dates or times [5].

### 1.3 Evolution of EMIs

Over the past decade, technology-delivered interventions have received considerable attention. Although text messaging EMIs can deliver psychological interventions in real-time, the introduction of smartphones has enabled a truly new type of EMI. A new class of EMIs, referred to as “just-in-time interventions”, adapt treatment delivery over time to provide interventions most likely to be effective, using information gathered through Ecological Momentary Assessment (EMA) [8] or sensing [9]. Even though EMIs and JITAIs share many of the same elements, JITAIs entail the provision of opportunistic interventions by adaptively improving and tailoring the interventions over time, and thus can be seen as a subset of EMIs. By 2023 the number of mobile broadband subscriptions is expected to reach 8.3 billion while the number of smartphone subscriptions is expected to equal about 70% of the world’s population [10].

A core feature of smartphones is that they are used in a variety of situations throughout daily life, and thus provide broad reach for the delivery of interventions. Furthermore, the powerful computational capabilities of smartphones, the introduction of sensors, and the ability to connect other wearable devices provides the opportunity not only to give support at opportune or high risk moments, but also to predict when they might occur.

In the meantime, the profileration of health apps, and mental health apps in particular has increased the need for research on mHealth interventions. As the seminal works on EMI were presented over a decade ago, it is now a timely moment to examine the literature on EMIs over the last decade to see how they are defined, and what they comprise in practice, as compared to the original vision.

This scoping review thus complements and extends the review of Heron & Smyth of 2010, which presented 27 studies, 9 of which were in the area of mental health, and none of which involved a smartphone app. We do this by considering work on mobile technology-based EMIs for mental health published since 2009, and exploring the design features of the interventions delivered.

### 1.4 Objectives

We articulate our objectives as research questions: (1) How are ecological momentary interventions defined in the literature on the treatment of common mental health problems? (2) What characterizes an ecological momentary intervention (EMI), and on what dimensions might we categorize EMIs? (3) Are they defined consistently across different application areas, and to what extent have the potential features been explored in these application areas?

A scoping review methodology using the framework presented by Arksey and O’Malley [11] and advanced by Levac et al. [12] was applied. A scoping review requires analytical reinterpretation of the literature and is commonly undertaken to clarify a complex concept and refine subsequent research inquiries [13].

## 2 Method

### 2.1 Eligibity criteria

Studies were included if they 1) were concerned with the prevention or treatment of a mental health disorder 2) concerned technology as part of the intervention or treatment delivery; 3) concerned momentary intervention or components of a momentary intervention; 4) were published in the English language.

Review articles regarding EMI were included if the studies they covered met the inclusion criteria, as they necessarily operationalize a definition of EMI, and provide an overview of perceptions of EMI within particular areas of mental health. Theoretical/analytic articles were included if they discussed EMI within the inclusion criteria. Studies were excluded if they 1) provided assessments without intervention; 2) examined interventions in a laboratory setting and not in real-world setting.

### 2.2 Information sources

In order to cover both clinical, psychological and computing research, this review was based on the PubMed, PsycINFO and ACM Guide to the Computing Literature databases. The selection of keywords for the search string related to mental health was informed by previous studies [5, 14, 15]. The search terms combined two main concepts: “momentary intervention” and “mental health” (a list of specific search terms are available in S1 Table). The initial search was carried out in June 2019, yielding 43 articles, and repeated using the same criteria in October 2020 in order to identify articles published since June 2019, resulting in an additional 21 publications, for a total of 64 articles. As the review is intended to complement and extend the review of Heron & Smyth and focus on EMI during the smartphone era, the search was limited to studies published between 2009 and 2020. The Heron and Smyth corpus included no smartphone apps; the first smartphone app in our corpus appears in 2011, and thus the review period covers the transition to smartphone app implementation.

### 2.3 Selection of sources of evidence

**Fig 1:**
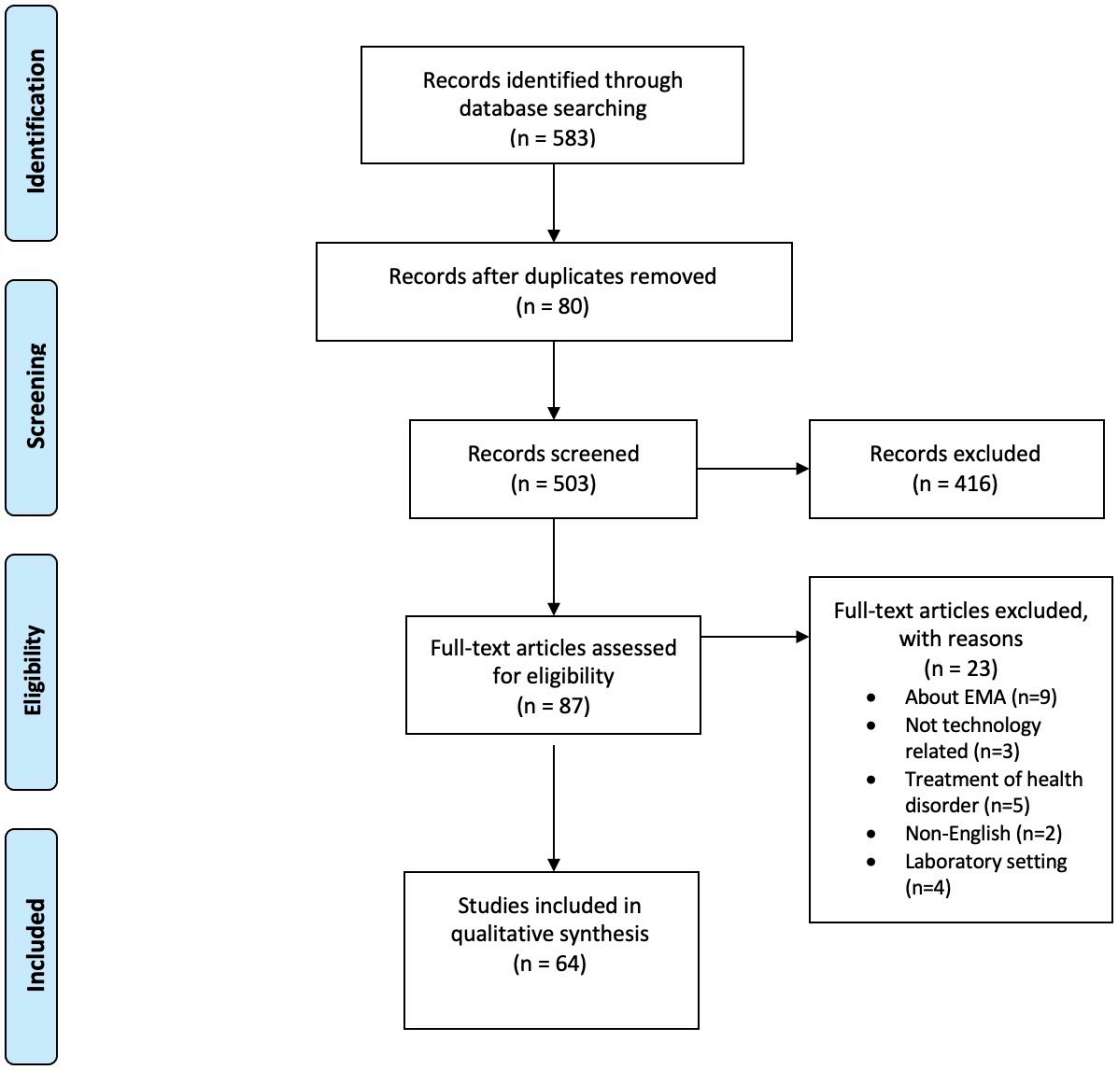
Flow diagram for paper eligibility.

The results from the search were imported into a Zotero citation manager where duplicate citations were removed and then exported into a spreadsheet for the screening process. A member of the research team independently screened all retrieved titles and abstracts for eligibility. A second author then reviewed a random sample of forty results to assess agreement and resolve any uncertainty about the inclusion or exclusion criteria. The study selection process is illustrated in Fig 1.

### 2.4 Charting, summarizing, and reporting the results of the review

Two authors agreed on the data extraction protocol. A standardized data extraction tool was developed and piloted by the first author, and refined based on feedback from a second author. One author then conducted the extraction. The data extracted comprised basic study information (author, year, location), sample characteristics (number of participants, age, gender), intervention aim, target of intervention, definition of intervention (if it is provided), platforms used for the delivery of the intervention, intervention strategies (CBT, ACT, etc.), components of intervention (psychoeducation, goal setting etc.), design/delivery of intervention components (discrete messages, multimedia pages, audio etc.), tailoring of intervention, timing of delivery, intervention period, human involvement. Human involvement was included as it is a powerful means of improving engagement [16], a mechanism by which treatment can be tailored to the individual, and supports deployment of EMI within higher intensity treatment. The model of Mohr et al. [17] was used to categorize sensors for automated monitoring, the features that are derived through data collected from sensors, and the prediction of behavioral markers and clinical targets.

The data were further coded under emergent themes using an iterative process. The coding scheme was created under the following categories: definitions of momentary interventions, components of ecological momentary interventions including devices used, information delivery, prompting times, sensors (if any used), feedback, training, tailoring, and support.

## 3 Results

### 3.1 Characteristics of source documents

In total 583 articles were identified in the database search, and following screening a final total of 64 articles were included in the review (see Fig 1 for details).

Of these papers, 42 studies described delivery of an intervention, 12 used sensors for the detection of mental health problems (with a specific stated aim to enable EMI) and 10 were reviews related to EMIs for mental health problems. S2 Table lists the characteristics of the studies included in this review.

Table 1 lists the mental health conditions targeted by those studies that involved delivery of an intervention, the intervention techniques employed. The studies’ general characteristics, type of technology, intervention delivery and intervention strategies related to each intervention are presented in S3 Table.

**Table 1:** Ecological momentary interventions.

Study	Target	Sample	Intervention strategy
Pramana et al. (2014)	Anxiety	Clinical	CBT
Kivity and Huppert (2016)	Anxiety	Clinical	Reappraisal
Pramana et al. (2018)	Anxiety	Clinical	CBT
Silk et al. (2020)	Anxiety	Clinical	CBT
Newman et al. (2014)	Anxiety (GAD)	Clinical	Cognitive therapy, relaxation
LaFreniere & Newman (2016)	Anxiety (GAD)	Clinical	CBT (worry outcome monitoring)
LaFreniere & Newman (2019)	Anxiety (GAD)	Clinical	CBT (worry outcome monitoring)
Wenze et al. (2014)	Bipolar Disorder	Clinical	CBT
Depp et al. (2015)	Bipolar Disorder	Clinical	Self-management strategies
Wenze et al. (2016)	Bipolar Disorder	Clinical	Cognitive behavioral principles
Burns et al. (2011)	Depression	Clinical	BA
Wahle et al. (2016)	Depression	Clinical	CBT
Levin et al. (2019)	Depression and anxiety	Non-clinical	ACT
Levin et al. (2019)	Depression and anxiety	Non-clinical	ACT
van Aubel et al. (2020)	Depressive psychotic complaints	Clinical	ACT
Juarascio et al. (2020)	Eating disorders	Clinical	CAT
Merkouris et al. (2020)	Gambling disorder	Clinical	CBT, motivational interviewing
Meinlschmidt et al. (2016)	Mood	Non-clinical	Mindfulness-based strategies
Meinlschmidt et al. (2020)	Mood	Non-clinical	Mindfulness-based strategies
Kroska et al. (2020)	Mood	Clinical	ACT
Stevenson et al. (2020)	Mood, alcohol use	Clinical	Coping strategies
Bell et al. (2018)	Schizophrenia	Clinical	Coping strategies (coping strategy enhancement) framework
Hanssen et al. (2020)	Schizophrenia	Clinical	CBT
Moitra et al. (2020)	Schizophrenia	Clinical	CBT
Pulantara et al. (2018)	Sleep disturbances	Clinical	Military-version brief BT for Insomnia (CBTI)
Versluis et al. (2018)	Stress	Clinical	Worry reduction training, mindfulness
Beute and Kort (2018)	Stress	Clinical	Exposure to natural scenes
Nguyen-Feng et al. (2019)	Stress	Clinical	Theory-based stress management intervention (by being the first to translate it into an EMI)
Lucas-Thompson et al. (2019)	Stress and anxiety	Clinical	Mindfulness
Dennis et al. (2015)	Substance use	Clinical	Self-determination theory
McTavish et al. (2012)	Substance use (Alcohol)	Clinical	Self-determination theory, cognitive-behavioral relapse prevention
Riordan et al. (2015)	Substance use (Alcohol)	Clinical	Psychoeducation about alcohol use and consequences
Leonard et al. (2017)	Substance use (Alcohol)	Clinical	CBT, Motivational interviewing
Dulin & Gonzalez (2017)	Substance use (Alcohol)	Clinical	Cognitive and behavioral alcohol use disorder interventions
Kazemi et al (2017)	Substance use (Alcohol)	No testing	Behavioral theory, MI, TTM of change
Moody et al. (2018)	Substance use (Alcohol)	Clinical	Active implementation intentions
Kazemi et al. (2019)	Substance use (Alcohol)	Non-Clinical	Motivational Interviewing
Haug et al. (2020)	Substance use (Alcohol)	Clinical	Implementation intention and action planning
Blevins et al. (2020)	Substance use (Alcohol)	Clinical	Coping strategies
Businelle et al. (2020)	Substance use (Alcohol)	Clinical	Motivational interviewing, self efficacy (SCT)
Shrier et al. (2018)	Substance use (Marijuana)	Clinical	Motivational enhancement therapy
Morgiève et al. (2020)	Suicide prevention	Clinical	Suicide prevention

### 3.2 Definitions

Twenty three of the studies cited the Heron & Smith definition of ecological momentary interventions [14, 15, 18–37]. Others provide their own definitions which are nevertheless consistent with the Heron and Smyth definition, such as “any technology-based device or application that can enhance care of patients … through the delivery of regular, momentary intervention in the context of daily life outside face-to-face therapy” [38].

A further sixteen studies used an implicit definition without clearly outlining and clarifying a definition for the interventions they deployed [39–54]. Three of these studies described delivery of *micro-interventions* to participants without providing a definition [55–57]. The micro-interventions delivered mindfulness-based strategies through video presentation and the delivery of an online daily diary for the practice of a cognitive reappraisal technique.

#### 3.2.1 EMA linked definitions

Several authors make an explicit link to EMA; a review that explored the feasibility, acceptability and clinical outcomes of EMA and EMI in the treatment of psychotic disorders, stated that “EMI is a derivative of EMA that extends the methodology of repeated within-environment prompting into the domain of clinical intervention” [38].

Another review of the use of EMA and EMI in the context of alcohol use refers to EMI as interventions that are delivered via mobile devices when the user needs it (i.e. in a high-risk situation) [58], an instantiation of the general point also made by Heron & Smith. The authors consider key characteristics of EMI as being the intervention aim and the timing of them. The review discusses the conjunction of EMA and EMI together since EMA can help inform the timing and content tailoring of EMIs [58]. The combination of ecological momentary intervention and assessment (EMAI) and its potential to intervene in high-risk situations, as stated by Beckjord et al., is mentioned in a study that examined the influence of smartphone-delivered in-the-moment coping strategies on drinking after experiencing a craving among participants utilizing an intervention [59].

Bell et al. also refer to EMI as an extension of EMA and refer to the notion of blended therapy [60]. Blended therapy is the combination of face-to-face and internet approaches for the delivery of an intervention [61]. While human involvement in the delivery of momentary interventions is possible, the notion of “ecological momentary intervention” is sometimes interpreted as minimizing or even substituting the role of human involvement.

#### 3.2.2 Just-in-time interventions

Even though it has been stated that EMIs and assessments run in parallel [5], the move towards the use of technology sensing and sophisticated algorithms to drive the delivery of interventions will change the role of real-time assessments. Thus, several studies discuss the potential of JITAI’s [34, 62–75].

As stated by Nahum-Shani et al., a just-in-time adaptive intervention (JITAI) is “an intervention design aiming to provide the right type/amount of support, at the right time, by adapting to an individual’s changing internal and contextual state” [9]. In a just-in-time adaptive intervention, the information gathered from a person or their environment are used to eliminate support that is not beneficial [9]. The distinguishing feature of just-in-time interventions compared to ecological momentary interventions is the use of statistical methods to improve and tailor the interventions over time for a given individual [24].

Foreseeable technology advances inspired both the original vision of EMI, and subclasses such as JITAI; thus we see that definitions emerge and evolve together with new technologies and components, and so it is appropriate to revisit and reconsider terminology as the technological ecosystem develops. In the following sections, we systematically describe the different components and characteristics of the EMI systems and studies included on this review.

### 3.3 Devices

Mobile technology offers the opportunity for the delivery of interventions in daily life and allows for the observation of participant behavior in daily life settings and contexts. As expected from the selected date range for the review, 39 out of 42 intervention studies used mobile phones as the main platform for the intervention deployment; the remaining 3 studies used a palmtop computer. Of these intervention studies, eight used mobile phones for the delivery of prompts to access the intervention content online through a web browser [25, 27, 28, 52, 53, 55–57], and four to deliver intervention content through SMS messages [26, 35, 46, 68]. The shift towards smartphone implementation is clearly seen in the corpus; 38 of 54 studies deployed mobile apps. More specifically, mobile apps were used for the delivery of an intervention in 30 studies, and the remaining 8 studies used an app for the collection of sensor data. One intervention delivered intervention content with the use of two different applications, each of them for the delivery of different intervention techniques [54]. Another study used an external application to measure cardiac activity [54]. Twelve studies additionally used a wearable device to collect measurements of bio-physiological [14, 41, 44, 65–67, 71, 74–76] and physical data [63, 64].

Two of the studies used a website to supplement individual CBT [19] and group CBT [20, 21], one to provide psychoeducation and feedback [40], one to assign additional interventions and monitor participant’s progress [22], one to assign additional interventions and set EMA schedules [63], and one to provide just-in-time support by the clinicians that reviewed participants entries on the mobile phone [64]. The widespread adoption of smartphones has made ecologically valid deployments easier to implement; twenty four of the studies delivered intervention content to smartphones that were owned by the participants [18, 21, 22, 30, 31, 33–35, 43, 44, 47, 48, 51, 52, 55, 56, 60, 62, 64–66, 68, 70, 71]. Five of the studies provided smartphones to participants if they did not own one [14, 39, 46, 49, 50]. Participants in twelve of the studies received wearable devices for the collection of sensor data [14, 41, 44, 63–66, 66, 67, 71, 74, 75].

### 3.4 Information delivery

The EMIs included in this review delivered a variety of intervention strategies to participants. Many of the studies combined different psycho-social intervention strategies to achieve their aim. The interventions used different types of technology for the delivery of intervention content including the use of messages, applications and additional treatment components such as websites.

#### 3.4.1 External delivery: Paper, SMS and web links

Paper-based exercises have long been used in mental health, and written exercises may have a role to play in EMI to avoid laborious text entry, or where participants are concerned about the privacy of electronic data. Two interventions in this corpus prompted participants with SMS messages to complete written exercises as part of the intervention delivery [52, 53]. Four interventions delivered intervention content online. Participants received text messages [28, 56] or invitation emails [55, 57] to access mobile web-based programs for the delivery of the interventions.

Several studies used messages for the delivery of intervention content through SMS [26, 35, 46, 68]. Two of these studies used both SMS and web links for delivery of intervention content [35, 68]. Novel uses of SMS included delivery of a quiz, and a contest involving creation of motivational text messages [68].

#### 3.4.2 Mobile phone applications

Intervention content was delivered via a native mobile phone app in 30 studies. Four of those studies provided a linear program, delivering the content to participants in a predefined manner [29, 54, 64, 67]. The remaining applications allowed participants to access the different intervention components at any time [19–22, 31–34, 36, 40, 44, 45, 47–51, 60, 62, 63, 65, 69–71, 77]. One intervention contained only one component [29] while the rest offered a variety of components to users.

There is often little information available about the rationale for the design of components, and the mechanisms for delivery of content. Therefore the following sections present findings from those studies that offered some description of the different modalities for intervention content delivery.

#### 3.4.3 Textual content

Text was used for the delivery of both psychoeducation [31, 36, 46, 65] and intervention strategies [32, 33, 48, 49, 60, 62]. For example, one of the interventions provided textual educational material spanning multiple screens (e.g. ‘On the following three pages, you will get an introduction to awareness’) [65]. Another intervention used a library component which provided participants with single pages of text on a range of skills, each conveying a strategy from acceptance and commitment therapy, and instructing them to practice it in the moment [62].

#### 3.4.4 Question-based delivery

Nine studies delivered psychoeducation and intervention strategies through the use of questions [19–21, 25, 36, 44, 54, 65, 67]. For example, psychoeducation was delivered with the use of a quiz element that asked questions about educational material shown to the user previously (e.g. “How do you define awareness?”) [65]. Another intervention, aiming to prevent relapse to heavy drinking, delivered psychoeducation with the use of frequently asked questions [44].

#### 3.4.5 Conversational interaction

One intervention integrated aspects of motivational interviewing into a virtual coach component which guided participants through an exercise to explore the pros and cons of making a change in personal alcohol use. The app engages in a “conversation” with the participant to elicit information and present strategies (e.g “Would you like to calculate your blood alcohol concentration from yesterday?”) [31].

#### 3.4.6 Multimedia content delivery

Several studies offered multimedia content to participants. Mindfulness and relaxation were typically offered through the use of audio and video. Six studies delivered breathing exercises with the use of video [20, 21], audio [54, 65], and audio or text [62]. Seven of the studies used audio recordings to deliver mindfulness based exercises [34, 54], relaxation [19–21], or other kinds of audio content [59, 62]. For example, a study comparing in-the-moment skill coaching effects from tailored to non-tailored ACT, included audio-guided (or text-based) exercises to practice skills such as acceptance of a difficult emotion, cognitive defusion, breathing mindfulness and reflecting on a valued moment [62]. Video was used to deliver psychoeducation [47] or to introduce and demonstrate the various features of an application [31, 63, 71]. One study delivered intervention content with the use of audio-visual components and minimal text [34].

Others focused on the use of visual images; for example, exposure to natural images through the use of slideshows delivered on the phone [29]. One of the studies displayed photos taken by participants to remind them of the reasons for deciding to change their drinking behaviors [59]. Video messages from therapists together with photographs and documents have been used to remind participants to practice skills learned during face-to-face sessions (i.e. “coping cards” completed during session) [19–21]. In another exercise, participants could see themselves on the smartphone, using the front camera in order to receive an instruction (e.g. “You will see yourself on the phone. Look yourself in the eye and smile for at least 20 seconds”) [65].

### 3.5 On-demand support

A distinct category of support feature relates to functionality that is used by clients when they feel they are particularly in need, or at risk (e.g. at risk of relapse) [32, 44, 49, 51, 63, 69]. One of the interventions integrated additional components to provide in-app and on-demand support with the provision of immediate help to avoid an imminent relapse, alerts to key people who may reach out to the participant, and recovery experiences [44]. Other studies offered in-app support by providing the options to contact a social network of relatives or a mental health professional [51], or by automatically contacting a list of contacts identified by the participants in case they travel to a gambling venue [69]. On-demand support was provided in two studies by integrating weekly exercises based on topics covered in previous therapy sessions [32], or by presenting intervention strategies previously selected by the participants [49].

Some interventions provide links to external resources outside of the intervention. Six of the studies in this corpus implemented a resources feature with links to external web resources [44, 46, 51] or through the app [31, 47, 63].

### 3.6 Prompting interaction with EMI interventions

The use of an EMI in daily life entails remembering to access the intervention. For that reason, when designing an EMI, participants are typically prompted to interact with the intervention during the day. We use the term ‘prompt’ to describe features that prompt users to interact with the EMI intervention.

Some form of prompting was described in 38 intervention studies. Prompting may occur at specific or random times determined before the delivery of the intervention, user-initiated times and at opportune moments based on sensor data collected during the intervention period (Table 2).

**Table 2:** Prompting of ecological momentary interventions.

Reference	Fixed times	Random times	User-initiated	Event-based
Pramana et al. (2014)	X	X		
Kivity and Huppert (2016)	X			
Pramana et al. (2018)		X	X	X
Newman et al. (2014)	X			
LaFreniere et al. (2016)		X		
Sarkeret et al. (2016)		X		
Versluis et al. (2018)		X		
Beute et al. (2018)		X		
Nguyen-Feng et al. (2019)	X			
Wenze et al. (2014)	X			
Depp et al. (2015)		X		
Wenze et al. (2016)	X			
Burns et al. (2011)		X	X	
Wahle et al. (2016)				X
Levin et al. (2019)	X			
Meinlschmidt et al. (2016)	X			
Wang et al. (2017)	X			
Bell et al. (2018)		X		
McTavish et al. (2012)	N/A	N/A	N/A	N/A
Leonard et al. (2017)				X
Dulin and Gonzalez (2017)	N/A	N/A	N/A	N/A
Riordan et al. (2017)	X			
Shrier et al. (2018)		X		
Kazemi et al. (2019)	N/A	N/A	N/A	N/A
Moody et al. (2018)	X			
Dennis et al. (2015)	X		X	
Levin et al. (2019)		X		
Stevenson et al. (2020)		X		
Hanssen et al. (2020)	X			
Haug et al. (2020)	X		X	
van Aubel et al. (2020)		X		
Moitra et al. (2020)		X		
Juarascio et al. (2020)	X		X	
Blevins et al. (2020)		X		
Silk et al. (2020)		X	X	X
Meinlschmidt et al. (2020)	X			
Morgiene et al. (2020)	N/A	N/A	N/A	N/A
Merkouris et al. (2020)		X		
Kroska et al. (2020)	X			
Businelle et al. (2020)			X	
LaFreniere et al. (2019)		X		
Lucas-Thompson et al. (2019)	X			

Fixed times: Prompt delivered at specific times; Random times: Prompt delivered at random times; User-initiated: Prompt delivered upon user preferences; Event-based: Prompt delivered based on the collection of sensor data

### 3.7 Gamification

Gamification refers to the use of game design elements in non-game contexts [78]. Four studies employed gamification techniques. One study deployed educational games with the use of multiple choice and true/false questions [31]. Another intervention familiarized participants with intervention content through a conversation via SMS between the children participating in the study and a virtual friend [20, 21]. All four studies offered points and rewards for completing assessment entries [19–21], or based on scores received when engaging in competitions with other participants [31]. While gamification is used to encourage client engagement, game metaphors may also be more deeply integrated with the therapeutic material.

### 3.8 Sensors for automated data monitoring

Twenty two of the studies used sensors to automatically collect data, provide sensor-based responses or attempt to predict psychological states. We use the hierarchical framework of Mohr et al. [17] to categorise the sensors used (Table 3), and the transformation of the sensor data into features for the prediction of behavioral markers (Table 4). S4 Table provides a list of the studies that used sensors.

**Table 3:** Sensors for automated data monitoring.

Study	Location	Movement	Phone screen	Phone apps	Ambient Light	Mic	Comms	Phys
Pulantara et al. (2018)		X						X
Wahle et al. (2016)	X	X	X				X	
Saeb et al. (2015)		X	X					
Chin-Lun Hung et al. (2016)							X	
Wang et al.(2017)			X	X	X	X		
Burns et al. (2011)			X	X	X			
Versluis et al. (2018)								X
Sarker et al. (2016)	X							X
Beute & Kort (2018)				X				
Wang et al. (2018)	X	X				X	X	X
McTavish et al. (2012)	X							
Leonard et al. (2017)								X
Bae et al. (2018)		X	X				X	
Pramana et al. (2014)	X							
Mishra et al. (2020)	X							X
Jacobson et al. (2020)	X						X	X
Rashid et al. (2020)	X	X					X	
Epstein et al. (2020)	X							
Coral et al. (2020)	X	X						
Kroska et al. (2020)								X
Businelle et al. (2020)	X							X
Kaczor et al. (2020)								X

Mic = microphone, Comms = in-phone communication, Phys = Physiological Sensors

**Table 4:** Features derived from data collected from sensors.

Study	Location type	Activity type	Movement	Phone usage	Bedtime	Paraling	Acoustic movement	Social
Pulantara et al. (2018)					X			
Wahle et al. (2016)		X		X				
Saeb et al. (2015)			X	X				
Chin-Lun Hung et al. (2016)				X				
Wang et al. (2017)				X	X	X		
Burns et al. (2011)	X	X						X
Versluis et al. (2018)								
Sarker et al. (2016)		X						
Beute & Kort (2018)				X				
Wang et al. (2018)		X		X	X	X		X
McTavish et al. (2012)	X							
Leonard et al. (2017)	X							
Bae et al.(2018)		X		X				X
Pramana et al.(2014)	X							
Mishra et al. (2020)	X	X						
Jacobson et al. (2020)	X		X					X
Rashid et al. (2020)	X	X	X					X
Epstein et al. (2020)	X							
Coral et al. (2020)	X	X						
Kroska et al. (2020)					X			
Businelle et al. (2020)	X							
Kaczor et al. (2020)	X							

Movement = movement intensity, Paraling = paralinguistic information, Social = in-phone social activity

#### 3.8.1 Sensor-based measurements

Three of the studies used sensors as part of the intervention in order to collect outcome measurements. The studies collected cardiac activity data with the use of a chest band worn underneath participants clothing [54], measured heart rate when participants held their fingers in front of the phone camera by using an external application (MyHeartRate) [29], or collected sleep, heart rate, and steps data through an activity tracker [71].

#### 3.8.2 Sensing for detecting and predicting states relevant to mental health

Twelve studies utilized sensors for the prediction or detection of psychological states. Sensors were used to detect stress episodes [66, 74, 79], negative emotions [18], substance use (i.e. high-risk drinking events) [43], predict subjective measures of social anxiety [73], stress and drug craving events [75], symptoms of schizophrenia [42], or depressive states [39–41, 72]. S5 Table provides an overview of the devices used and the collected data from sensors for the prediction of each mental health condition.

#### 3.8.3 Sensor-based responses

Seven studies attempted to tailor intervention content based on data collected by sensors.

*3.8.3.1 Activity based responses*. One study incorporated a variety of sensors for data collection that were analyzed and transformed into context information that in combination with user preference and decision logics were used to recommend evidence-based interventions presented via the application. For example, when a low activity level was detected by the system, participants were recommended to take a walk [65].

*3.8.3.2 Location based responses*. Four of the studies used geofencing to complement time-based reminders and provide support to the participants [20, 44, 63, 69]. Just-in-time prompting to access intervention content was initiated based on locations predefined during a face-to-face session with a therapist [20], registered by the participants through an app [44, 69], or determined based on assessment responses [63]. One of the studies used GPS to collect geofencing data when an internet connection was enabled and implemented an algorithm using phone sensor data (gyroscope, accelometer, and magnetometer) to detect location when an internet connection was not available [69].

*3.8.3.3 Physiologically based responses*. Two of the studies used physiological data to provide interventions [64, 67]. The studies used wearable devices to measure sleep-wake patterns [64] or electrodermal activity (EDA) [67] to decide when to prompt users to interact with personalized recommendation or intervention content.

### 3.9 Feedback

Feedback can be important for maintaining engagement by participants; 25 studies delivered feedback messages to participants as part of the intervention.

#### 3.9.1 Motivational messages

Five of the interventions delivered motivational messages to participants after the completion of assessments [19, 30, 35, 47, 55]. For example, in a RCT study aiming to reduce marijuana use in youth, participants received motivational messages via the application after the completion of a momentary report of personal triggers for marijuana use, desire, use or effort to avoid use. Where their response rate was low, the messages reminded participants to respond and advised them to seek technical support [30]. Another intervention sent one motivational message daily that was drawn from a pool of messages based on motivational interviewing techniques, mindfulness-based therapy approaches and encouraging language found in other motivational messaging EMIs [47].

#### 3.9.2 Semi-individualized feedback

Seventeen of the studies provided semi-individualized feedback with the use of branching rules that were programmed into the software, and based on EMA responses [22, 25, 27, 32–36, 40, 48, 50, 51, 62, 63, 67, 68, 71]. Feedback included the delivery of intervention strategies or encouragement messages based on the answers provided during check-in assessments. For example, in one intervention participants, with the use of CBT-informed questions, provided their current emotions and level of intensity, and current context. When a positive emotion was identified they received an affirmational statement; a coping strategy was suggested when they selected a negative emotion. Additionally, when they selected a negative emotion, they were asked about their intentions to drink alcohol and their current context in order to receive specific coping strategies [67].

#### 3.9.3 Graphical

Nine of the interventions provided data visualization of past entries [19–22, 28, 31, 40, 64, 71]. Five of the interventions allowed therapists to monitor participant entries through the integration of a web-based portal which displayed a graphical representation of participants entries [19–22, 64]. Three interventions provided feedback to participants to identify changes over time [28, 31, 71]. Another intervention provided graphs through a website for a variety of purposes such as progress over time, exploration of emotion changes based on location data, and identification of patterns based on graphs created by a therapist [40].

### 3.10 Tailoring

The tailoring of interventions can be categorized into three groups based on the level of human involvement needed for the delivery of personalized content:

#### 3.10.1 Manual tailoring

We define manual tailoring as where a participant, a clinician or other professional configures the EMI behaviour for participants. Clinicians performed tailoring in eleven of the studies. This has been achieved through use of a clinician portal. For example, such a portal has been used to monitor patient adherence, manage rewards points, send audiovisual material to patients, customize instructional messages, and send customized motivational messages [19–21]. Additionally, clinicians performed tailoring by selecting intervention strategies [22, 60, 63], and setting the time ranges for participants to be notified [19–21, 63].

Research assistants and therapists in two studies that used sensors as part of their strategy offered manual tailoring during the intervention period. This was done by setting a threshold to alert participants when EDA was high [67], or by predefining rules to prevent unreasonable intervention recommendations by an application that used a learning system to deliver appropriate interventions depending on participants context [65].

In three of the studies participants selected intervention content during face-to-face sessions, which was then integrated into the mobile application [28, 32, 60]. The intervention content selected during those sessions was presented based on mood scale ratings [28, 32] or based on a predefined plan for the intervention [60].

Two of the studies offered the option to modify the content and order of suggestions provided through the app [51] or to upload pictures/videos and sounds to customize future visual and auditory alerts [69].

#### 3.10.2 Semi-individualized tailoring

Semi-individualized tailoring involves automated selection of interventions, common across participants, that are selected based on participant preferences. Eight of the interventions delivered EMIs based on participant preferences before the delivery of the intervention [31, 33, 46, 48, 49, 51, 68] or based on clinician decisions [64]. One study populated a matrix with ACT-based interventions on the home screen of the app based on assessment responses [71].

Sensor data may be used to drive tailoring, as in the case of the intervention aiming to prevent drinking relapse mentioned previously, where participants voluntarily registered places they consumed alcohol and based on GPS data the system provided just-in-time support [44].

#### 3.10.3 Automated tailoring

We define automated tailoring as tailoring which does not involve any configuration input from clinicians or clients. One intervention aiming to deliver CBT for people with depression automatically presented interventions to users based on sensor and phone usage data. The application made recommendations based on a variety of features including time of the day, location, smartphone usage and physical activity data [65].

### 3.11 Training

Training often forms part of the delivery of ecological momentary interventions. In 27 of the studies included on this review, research staff and clinicians offered training to participants before the delivery of the interventions for a variety of purposes, including instructions for use of intervention content, and the use of the technology [20, 21, 27–30, 33–36, 39, 40, 42, 47–53, 55, 56, 60, 62]. Three of the studies delivered video tutorials of the intervention after downloading the app [31, 63, 71].

### 3.12 Human support

The involvement of research staff and clinicians is not restricted to the tailoring of interventions. Support is provided during the intervention period for a variety of reasons.

#### 3.12.1 Support between treatment sessions

Support was provided in six studies where EMI was delivered in parallel with face-to-face treatment sessions with therapists. In one of those studies, the therapist used the information gathered from the intervention to gauge client progress [36]. Four interventions offered support between face-to-face sessions through the EMI platform to provide feedback to participants upon request, and to select intervention strategies to be delivered via the app [19–21, 32]. Therapists in one study offered technical support during face to face sessions and integrated intervention content into the app in between sessions [22].

#### 3.12.2 Therapist initiated remote support

In fourteen studies, support was provided during the intervention period from research staff and clinicians without the use of face-to-face meetings with participants. This often involved therapists calling participants to address any technical problems with the software and hardware [25, 27, 28, 34, 39, 49, 63, 67] or to support adherence by calling participants when necessary [40, 50, 56]. In three studies, support was provided to participants who reported a deterioration of symptoms based on answers derived from EMA responses [42, 64], or when accessing a component of the app when in need of help [51].

#### 3.12.3 In-app human support

Professional and peer support was provided to participants through the integration of a variety of features into the applications deployed. Five studies integrated support directly into the applications.

Therapist support was provided in four studies through the integration of features that allowed participants to compose messages on the phone that were sent to a portal administered by therapists [19–21], or by offering the ability to receive personal responses to questions from experts in addiction within 48 hours through SMS messages [44].

Peer support was offered in two studies [44, 59]. One of the studies implemented discussion groups where participants could anonymously exchange emotional support and information with others [44]. Another intervention offered in-app peer support through components that provided text or phone contact details of a person selected by the participants, or a link to their smartphones contact list to contact a friend or family member [59].

## 4 Discussion

This paper aimed to review the definitions used across the literature to describe EMIs for mental health disorders, and to categorize the design options available for the deployment of these interventions.

### 4.1 Defining EMIs

The vision of Patrick et al. [6] to deliver tailored ecological momentary interventions based on ecological momentary assessment responses became feasible a decade ago, as demonstrated in the synthesis and critique of technology-based EMIs by Heron & Smyth [5]. In their review, they provided a concise definition for the term as “treatments that are provided to people during their everyday lives (i.e. in real-time) and in natural settings (i.e. in real-world)”. Twenty three of the papers in our corpus used that definition and another sixteen provided an implicit definition by delivering an intervention that they described as an EMI. We thus must consider the components of these interventions, and in this regard we have seen that a modern EMI typically entails a range of information delivery, engagement and communication features.

Bell et al., in this corpus, provided a more precise definition of EMI as “any technology-based device or application that can enhance care of patients … through the delivery of regular, momentary intervention in the context of daily life outside face-to-face therapy” [38]. Even though this definition describes the ecological aspect of an intervention in more detail, the notion of *momentary* interventions is not defined as clearly. A decade ago, the “momentary” aspect was restricted to delivering interventions at random and scheduled times [5]. The concept of “just-in-time interventions”, which use statistical methods, and algorithms for the optimization of the time of delivery of individual interventions [24], has expanded the momentary aspect of EMI definitions, and fifteen studies in this review refer to the notion of “just-in-time” interventions.

As noted in the Definitions section, several authors discuss the conjunction of EMA and EMI in EMAI systems, and wrote about the importance of EMA to inform the timing and content tailoring of EMIs and thus intervene in high-risk situations [58, 59]. As EMA alone may have a therapeutic effect, extending EMA through the introduction of sensing capabilities may further blur the distinction between EMA and EMI. There was no consistency in the definition used, if any, among application areas. Multiple definitions may also apply to a given intervention; the development of JITAI offers the potential for altering the role of ecological momentary assessments in the future, and hence while not all JITAI are EMAI systems, some are.

We have seen in this review that key characteristics in the design of these interventions include the set of intervention components, decision mechanisms regarding when and which interventions should be provided to users, tailoring variables, and decision rules that link tailoring variables to intervention options. As the development of these components continues, the variety of approaches to tailoring and use of sensor data motivates further, more specific terminology to provide clarity for an understanding of the different characteristics deployed in any intervention given among different application areas. This terminology should convey the characteristics of an intervention including (a) the use of momentary assessment technology, (b) the use of sensors, (c) the involvement of professionals, and (d) the use of just-in-time algorithms that adapt the provision of support over time based on time-varying (dynamic) information collected from the user. Future studies should explicitly specify in their terminology these different aspects used for the deployment of their interventions and provide a rationale for the different components integrated into them.

### 4.2 EMI in the smartphone era

Mobile apps are now the most common medium used for the delivery of EMIs. This change has created opportunities for the use of multimedia as a medium for presentation of different intervention strategies and the deployment of more interactive interventions. Furthermore, mobile phone capacities have allowed for the integration of techniques such as gamification in order to enhance the experience of users. The expansion of technology offers the possibility of providing support not just with the incorporation of additional intervention channels such as interactive websites [5], but also with the integration of communication features. This extends the channels through which support can be provided and expands the options available for social support and enabling communication between individuals and clinicians. Furthermore, the advance of technology has allowed researchers to utilize sensors embedded on phones or wearable devices for the collection of environmental or other contextual data.

### 4.3 Sensing, machine learning and EMI

Patrick et al. envisioned the collection of environmental data or other contextual factors for the delivery of ecological momentary interventions in daily life. Heron & Smyth referred to the possibility of tailoring momentary health behavioral messages with the use of assessments of physiological or environmental cues as technology continues to become more sophisticated. The approach of delivering intervention content based on unobtrusive data collection has become feasible in recent years. Researchers are using sensors embedded on mobile phones and wearable devices to collect data for the provision of just-in-time support and prediction of mental health states, with 10 of the intervention studies incorporating sensor based components, and a further 12 studies focused on development of such components. Machine learning algorithms have been used to identify and predict a variety of states relevant to stress, depression, schizophrenia, and alcohol abuse. We have also seen that the use of sensors can enable opportunistic delivery timing by providing support when the participants are in need of support.

Two previous reviews emphasize the importance of tailoring interventions based on individual needs [80, 81]. Truly individualized tailoring requires a more sophisticated technology implementation, and research has explored various levels of human involvement in tailoring. The use of machine learning models offers the possibility for systems to make decisions with reduced human involvement, which carries with it a new set of concerns, and must be approached carefully [82]. Perhaps surprisingly, all studies that targeted depression used machine learning to identify depressive states.

These attempts to integrate machine learning models highlight a promising area for future development; that as technology advances we will be able to enhance the “momentary” aspect of the interventions by delivering interventions at event-based times and presenting content relevant to the person’s context.

### 4.4 Maturity of the field and clinical validation

The EMIs delivered a variety of different intervention techniques and most of the interventions combined different psycho-social strategies to achieve their aims. The literature, while growing in size and significance, can thus be seen as still constituting a very preliminary investigation of the topic.

The majority of studies focused on delivering interventions for anxiety and substance use disorders, with major topics in mental health unaddressed; for example, only one study was found that targeted an eating disorder, consistent with previous review findings [23]. This scoping review includes a variety of experimental design approaches, ranging from a (n = 3) case study to randomized controlled trials (RCTs). Nine RCTs of EMIs are included in this review, for anxiety, stress, bipolar disorder, substance use (i.e. alcohol), mood and depression. Of these RCTs four used EMA (without EMI) as a comparison group, one used CBT, one compared EMI with a paper and pencil condition, and two compared two different versions of the application in addition to an EMA only condition. Randomized controlled trials are the gold standard for evaluating interventions since they might tell us what treatments are beneficial. However, traditional two-armed RCTs can not provide insights about the timing, type and frequency of different interventions. An alternative promising methodology to address these issues when evaluating EMIs is the use of microrandomized trials (MRTs) to optimize treatment and to compare interventions to each other and to no intervention [24].

### 4.5 Recommendations for future research

The studies in the corpus provided little information about the design choices for the deployment of the interventions beyond describing (often briefly) the delivery platform. We therefore recommend that researchers present the rationale for choices made when designing an intervention in order to provide an understanding for the usefulness of different components deployed on them. This further raises the question of evidence for the effectiveness of different intervention components and different intervention designs. The studies targeted a variety of mental health disorders and deployed a variety of intervention techniques. This suggests the need to explore the effectiveness of different technological and interaction possibilities designed for specific mental health conditions and intervention techniques.

### 4.6 Strengths and limitations

To the best of our knowledge, this is the first review to present an overview of the different definitions used to describe ecological momentary interventions, and the first attempt to categorize the design options available for the deployment of these interventions. However, there are limitations in the interpretation of these findings. A quality appraisal was not included in the aims of this review. It is also worth noting that this was a scoping review of the literature rather than a quantatitve synthesis of EMIs, such as a meta-analysis would provide. Given the advances of this field in recent years, it is worthwhile to understand what is being done and how EMIs are being conceptualized. Clarity on these aspects might help in the development of standardized strategies for delivery, and facilitate future quantitative synthesis. This review focused on the delivery of interventions for people with mental health disorders; examining other work within mobile health interventions generally would also be valuable.

## 5 Conclusion

With the introduction and widespread adoption of smartphones, the literature has begun to realise the ambitious vision of EMIs. EMI implementation has shifted definitively towards smartphone app implementation, supporting a range of delivery modalities and engagement and communication features. The research literature has begun to explore the capabilities of both sensing and machine learning in the delivery of momentary interventions, as well as a variety of engagement features enabled through the use of smartphones. Exploitation of data from EMA has also been a key topic for researchers in the area. Clinical validation of these systems is still in the early stages, and a particular problem is the many design parameters involved in EMI delivery, concerning the timing and content of interventions. While the term JITAI has been introduced to describe the subset of EMIs with more sophisticated targeting of interventions, important design dimensions such as human involvement and the type of tailoring are not captured in existing terminology.

## Supporting information

S1 Checklist Preferred Reporting Items for Systematic reviews and Meta-Analyses extension for Scoping Reviews (PRISMA-ScR) checklist. (DOCX)

S1 Table Search string. (XLSX)

S2 Table List of included papers. (XLSX)

S3 Table Characteristics of ecological momentary interventions. (XLSX)

S4 Table List of studies using sensors. (XLSX)

S5 Table Studies using sensors to predict/detect psychological states. (XLSX)
